# Development of a new sandwich ELISA for the detection of bovine A1 beta-casein

**DOI:** 10.1371/journal.pone.0345548

**Published:** 2026-04-09

**Authors:** Ayumi Watanabe, Tomoe Kobayashi, Anna Okamoto, Daiki Oka, Tomohiro Noguchi, Ren Ozawa, Koumei Shirasuna, Makoto Matsuyama, Takashi Kuramoto

**Affiliations:** 1 Laboratory of Animal Nutrition, Department of Animal Science, Faculty of Agriculture, Tokyo University of Agriculture, Atsugi, Kanagawa, Japan; 2 Division of Molecular Genetics, Shigei Medical Research Institute, Okayama, Okayama, Japan; 3 Department of Agricultural Chemistry, Faculty of Applied Biological Sciences, Tokyo University of Agriculture, Setagaya, Tokyo, Japan; 4 Laboratory of Animal Reproduction, Department of Animal Science, Faculty of Agriculture, Tokyo University of Agriculture, Atsugi, Kanagawa, Japan; North-Caucasus Federal University - Pyatigorsk Campus: Severo-Kavkazskij federal'nyj universitet Patigorskij institut filial, RUSSIAN FEDERATION

## Abstract

The bovine genetic variant A2 beta-casein is associated with fewer digestive and absorption issues compared to A1 beta-casein, leading to increased global demand for A2 milk. However, contamination with the A1 variant during collection, transportation, or sterilization of A2 milk poses a risk, necessitating a verification test to ensure A2 milk does not contain A1 beta-casein. We developed an A1-specific monoclonal antibody (mAb) and a general mAb that reacts with both A1 and A2 variants, using the iliac lymph node method. A sandwich ELISA was created using the general mAb as the capture antibody and the A1-specific mAb as the detection antibody to identify A1 beta-casein in milk. This ELISA successfully detected A1 beta-casein in raw and pasteurized A2 milk, including ultra-high temperature treated milk. The test identified A1 beta-casein when the A1 spike in A2 milk exceeded 1% in volume, indicating its capability to detect contamination from one A1A1 cow in a herd of one hundred A2A2 cows. The developed A1 beta-casein ELISA is suitable for high-throughput analysis and valuable for monitoring A1 beta-casein contamination in commercially produced A2 milk.

## Introduction

Beta-casein, one of major milk proteins, exists in two main variants: A1 and A2. These variants arise from a single nucleotide polymorphism (SNP, rs43703011) in exon 7 of the bovine beta-casein (*CSN2*) gene, where cytosine in the A2 variant is replaced by adenine in the A1 variant. This substitution changes the codon from CCT (proline) to CAT (histidine) at position 67 of the protein [1]. Owing to this amino acid substitution, the A1 variant is thought to be processed differently from the A2 variant during digestion. Digestive enzymes commonly cleave both A1 and A2 at position 61; however, cleavage at position 67 occurs only in A1, not A2. Consequently, a seven-amino-acid peptide known as beta-casomorphin-7 (BCM-7), an opioid-like peptide, is released from A1 but not from A2 [2–4]. Because both A1 and A2 alleles are common in Holstein cow population [5], conventional milk contains both A1 and A2 variants.

Bovine BCM-7 has been shown to be a risk factor for cardiovascular disease, type 1 diabetes, sudden infant death syndrome, and neurological disorders such as autism and schizophrenia [2,6,7]. BCM7 is also involved in intestinal physiology, such as mucin release at the intestinal lumen [8], activation of immune-inflammatory cells [9], and IgA secretion [10]. These findings suggest adverse effects of A1 beta-casein on human health because of its ability to release BCM-7. To prevent such adverse effects, efforts have been made to produce A1-free A2 milk.

Scientific works related to A2 milk have largely focused on milk intolerance and gastrointestinal (GI) symptoms. Although a systematic review pointed insufficient human-based evidence to prove the adverse digestive effects of A1 compared with A2 milk [11], more recent studies have found that A2 milk decreases GI symptoms such as gastrointestinal inflammation [7], rapid gastric emptying [12], and prolonged digestive discomfort in lactose intolerant people [12,13]. Even in healthy toddlers, A2 milk improved overall digestive comfort and GI-related symptoms [14]. These findings suggest that A2 milk can improve the symptoms similar to lactose intolerance.

The potential positive impact of A2 milk on human health has globally promoted to produce A2 milk. Herds consisting of only A2A2 cows have been established in various countries such as New Zealand [15], Brazil [16], Mexico [17], Turkey [18], Italy [1], Poland [19], and Japan [20].

However, some challenges have occurred in producing A2 milk. The first was the effective selection of A2A2 cows from ordinary herds consisting of A1A1, A1A2 and A1A2 cows. To address the issue, we developed the easy and efficient beta-casein genotyping method [20]. The second challenge has been managing risk to contamination of A1 beta-casein in A2 milk. In the processing of milk from dairy farms to markets, milk collected from individual cows is typically stored in bulk tanks, transported by milk collection trucks, and sterilized and bottled in milk processing plants. During such process, contamination of A1 in A2 milk can occur, potentially leading to unintentional mislabeling. For example, a study in Austria found that four out of five samples labeled as A2 milk contained A1 beta-casein [21]. Thus, verification of purity of A2 milk, absence of the A1 variant, has been required.

Assays for detecting the A1 variant in milk have included HPLC-MS/MS [22,23], LC-MS [24], and ELISA [25,26]. While HPLC-MS/MS and LC-MS provide high analytical precision, they require complex sample preparation and specialized instrumentation, making them less practical for routine testing. In contrast, ELISA offers a more accessible and cost-effective approach, provided that antibodies specific to the A1 variant are available. Several A1 beta-casein ELISA assays have been developed [25,26]; however, these systems rely on polyclonal antibodies and require hazardous reagents for sample preparation. Moreover, their detection limit is approximately 5%, which is insufficient for verifying the purity of A2 milk with high sensitivity.

To address these limitations, we aimed to generate a mAb specific to A1 beta-casein, as mAbs generally provide higher reproducibility and accuracy than polyclonal antibodies. Due to their high specificity, an ELISA incorporating such a mAb is expected to achieve greater sensitivity than existing ELISA systems.

## Materials and methods

### Milk samples for sandwich ELISA

Individual milk samples were provided by a dairy farm in Furano, Hokkaido, Japan. They were collected from cows with known beta-casein genotypes (n = 67). Among them, 22 milk samples were defatted and used for antibody specificity testing, 35 were used in raw and 10 were used for sterilization. For defatting, 25 mL of milk was manually stirred for 1 h and centrifuged at 3,000 rpm at 4 °C for 15 min. The supernatant was filtered through 5A filter paper (Toyo Roshi Kaisya, LTD., Chiyoda, Tokyo, Japan), diluted 1:100 with phosphate-buffered saline (PBS), and stored at −80 °C.

### Pasteurization and ultra-high temperature processing

Pasteurization of milk was performed by heating at 63°C for 30 min. UHT processing was performed by heating at 121°C for 2s using a plate heat exchanger (HAS-CH-360, IWAI KIKAI KOGYO CO., LTD. Tokyo, Japan).

### Production of monoclonal antibodies

Animal experimentation was approved by Shigei Medical Research Institute Animal Care Committee (Approval Number: 22001). mAbs were produced using the iliac lymph node method [27]. Briefly, the A1 peptide (67His) with the sequence NH_2_-CFPGPIH^67^NSLP-COOH (Eurofins Genomics, Tokyo, Japan) was conjugated to keyhole limpet hemocyanin and used for immunization of BALB/cJ mice (The Jackson Laboratory Japan, Inc., Yokohama, Japan). The treated mice were sacrificed 21 days after the injection, and the lymphocytes were fused with SP2/0-Ag14 myeloma cells. After the cell fusion, culture supernatants were screened to select clones which reacted with the 67His but not with the 67Pro (NH_2_-CFPGPIP^67^NSLP-COOH) by solid-phase ELISA as described previously [27]. Among positive clones, we isolated clones that reacted to A1 but not A2 defatted milk diluted 1:10000 with PBS. The A1 and A2 defatted milk were prepared from A1A1 (n = 1) and A2A2 (n = 1) cows raised in a dairy farm in Fujinomiya, Shizuoka, Japan.

The mAb that reacts with both A1 and A2 was generated in WKY/NCrl rats (CLEA Japan, Inc., Tokyo, Japan) in a similar manner using a peptide common to A1 and A2 as the antigen. The sequence of the peptide was NH_2_-CESLSSSEESITRINKKIEK-COOH which corresponds to the residues from 29 to 47 of the bovine beta-casein [28].

### Milk protein preparation for Western blotting

Raw milk was defatted by centrifugation, filtered, freeze-dried, and stored at −20 °C until use. For extraction, 0.2 g of freeze-dried milk powder was dissolved in PBS (pH 7.4) containing 0.5% SDS and 2% 2-mercaptoethanol, mixed overnight, and centrifuged. [29]. The supernatant was filtered through a 0.8 μm membrane filter (AGC TECHNO GLASS Co., Ltd., Shizuoka, Japan). Bovine purified beta-casein (C6905, Sigma-Aldrich, St. Louis, MO, USA) was dissolved in 0.1 M Tris-HCl (pH 8.0) and used for the positive control.

### Western blotting

Western blotting was performed as described previously [30]. Briefly, Milk protein extracts (4.2 μg) from A1A1 and A2A2 samples, together with the purified beta-casein, were separated by 15% SDS-PAGE and stained with Coomassie Brilliant Blue (CBB Stain One, Nacalai Tesque, Inc., Kyoto, Japan). Protein-transferred membranes were blocked with 5% BSA and incubated overnight at 4 °C with either the general mAb or the A1 mAb (both diluted 1:3000 in blocking buffer). The secondary antibody was an HRP-conjugated goat anti-mouse IgG (Fortis Life Sciences, Boston, MA, USA; 1:3000) or an HRP-conjugated goat anti-rat IgG (Abcam, Cambridge, UK; 1:10,000). Signals were developed by Clarify Western ECL substrate (Bio-Rad Laboratories, Hercules, CA, USA) and detected with an ImageQuant LAS 4000 analyzer (GE Healthcare Japan Corporation, Tokyo, Japan).

### Sandwich ELISA

One μg of the capture antibody (general mAb) in 100 μL of PBS was added to each well of the Nunc-Immuno Plate (Thermo Fisher Scientific K.K., Tokyo, Japan) and incubated overnight at 37°C. Then, 200 μL of blocking buffer (1% BSA/PBS) was added and incubated at 37°C for 1h. Next, 100 μL of milk sample diluted to 1:10000 with PBS was added and incubated at 37°C for 2h. The detection antibody (A1 mAb) was added at 0.1 μg/100 μL in 1% BSA/PBS and incubated at 37°C for 2h. Goat anti–mouse IgG2a-HRP enzyme–labeled secondary antibody (Bethyl Laboratories, Montgomery, TX, USA) was diluted to 1:20000 with 1% BSA/PBS, and 100 μL of the diluted secondary antibody was added to the sample and incubated at room temperature for 1 h. Finally, 150 μL of developing solution consisting of 0.04% *o*-phenylenediamine (FUJIFILM Wako Pure Chemical Corporation, Osaka, Japan) and 0.04% H_2_O_2_ were added and incubated at room temperature for 30–60 min. The reaction was stopped by addition of 50 µL of 3M H_2_SO_4_. OD value was measured using a plate reader (Tecan Japan, Kawasaki, Japan) at a wavelength of 492 nm. Before adding each reagent except for H_2_SO_4_, the solution in wells was discarded and the wells were washed 2–3 times with PBS.

### Determination of detection limits

The detection limit for A1 beta-casein was determined using a discriminative point approach [31]. We combined the individual A2A2 milk (n = 5) equally to prepare the A2A2 milk mixture and added individual A1A1 milk to the mixture in which A1A1 milk was contained at 1%, 2%, 3%, 10%, 25%, and 50% milk spike, respectively. Along with 100% A1A1 and 100% A2A2 milks, these mixed samples were subjected to the ELISA in triplicate. Test mixtures of pasteurized and UHT milks were prepared in the same manner.

Cut-off value is calculated using the following equation:

$$\text{Cut}-\text{off }=\;\overset{―}{X}+\text{ SD}f$$

where $\overset{―}{X}$ is the mean of OD values of negative controls, SD is the standard deviation, and *f* is the multiplier which is derived from the critical values for a one-tailed *t*-distribution and obtained from *t*he number of negative controls and the confidence level [31]. In the present study, we obtained the *f* value at 95% confidence level.

When the OD value was lower than the cut-off, the mixture was considered negative for A1 beta-casein. When the OD value was equal to or higher than the cut-off, the mixture was considered positive for A1 beta-casein.

### Statistical analysis

Statistical comparisons between experimental groups were performed using one-way ANOVA. Post-hoc testing was conducted using the Mann–Whitney U test. All analyses were performed using the EZR software package [32]. A *P*-value < 0.05 was considered statistically significant.”

## Results

### The monoclonal antibody specifically detected A1 beta-casein

We obtained a rat mAb raised against a peptide common to both A1 and A2 beta-casein, “general mAb”, and a mouse mAb raised against the A1-specific peptide (67His), “A1 mAb”.

**Fig 1:**
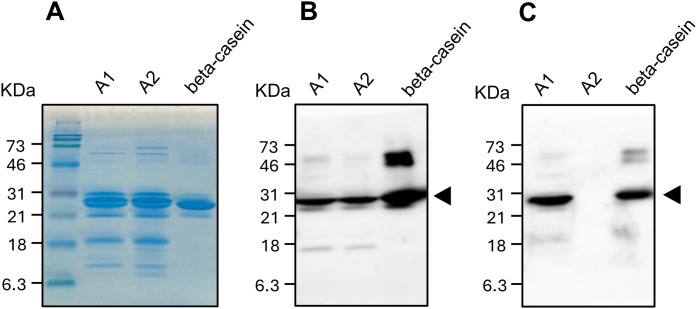
Western blot analysis of milk protein extracted from A1A1 and A2A2 milk. A, Proteins separated on SDS-PAGE gel were stained with Coomassie Brilliant Blue. B, Western blotting analysis with the general mAb that was raised against the peptide common to A1 and A2 beta-casein. This mAb detected 21–31 kDa bands from both proteins extracted from A1A1 and A2A2 milk as well as from the purified bovine beta-casein (arrowhead). Unintended bands (46–73 kDa) found in the purified beta-casein was likely derived from self-association of the beta-casein.

To examine specificity of the mAbs, we performed Western blotting analysis with the extracted milk protein and the commercially-available purified beta-casein that was obtained from ordinary milk containing both A1 and A2 (Sigma-Aldrich). The general mAb detected protein bands in the 21–31 kDa range in every sample, which corresponded to the beta-casein (Fig 1B). In contrast, the A1 mAb detected a 21–31 kDa band in the A1A1 milk protein and the purified beta-casein but not in the A2A2 milk protein (Fig 1C).

The A1 mAb is very likely to recognize an epitope located within the 67His peptide. To assess potential homology with other caseins, we performed a BLASTP search using the 67His peptide sequence (NH_2_-CFPGPIHNSLP-COOH). The BLASTP results indicated that the peptide exhibits significant similarity exclusively to beta-casein and not to other caseins (alpha s1-, alpha s2-, or kappa-casein) (S1 Table). Thus, we consider it highly unlikely that any epitope within alpha s1-, alpha s2-, or kappa-casein could be recognized by this antibody.

### The sandwich ELISA was applicable to raw and sterilized milk

**Fig 2:**
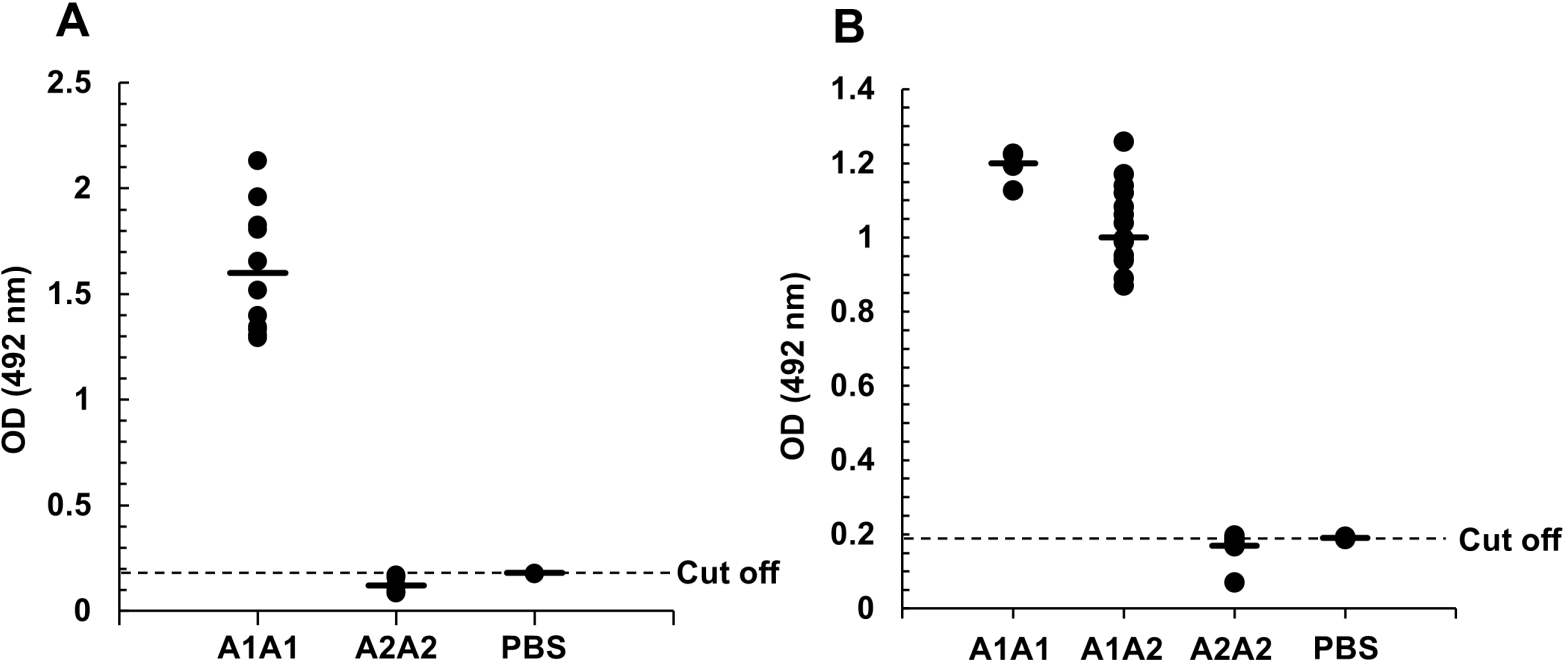
Distribution of OD values obtained from ELISA. A, OD values were obtained from A1A1 (n = 11) and A2A2 (n = 11) with defatted milk samples. The horizontal dashed line represents the cut-off value calculated from the OD of PBS (0.18 ± 0.0005). As the multiplier factor (*f*) was 3.37 at 95% of confidence level, the cut-off value was calculated as 0.177. B, OD values were obtained from A1A1 (n = 4), A1A2 (n = 13), and A2A2 (n = 18) with raw and sterilized samples. The horizontal dashed line represents the cut-off value calculated from the OD of PBS (0.19 ± 0.003). As the multiplier factor (*f*) was 3.37 at 95% of confidence level, the cut-off value was calculated as 0.200.

To examine the specificity of the mAb in milk, we performed sandwich ELISA using the general mAb as a capture and the A1 mAb as a detection antibodies. Optical densities (OD) of A1A1 defatted milk (n = 11) were all higher than the cut-off value (0.177) and the mean OD was 1.56 ± 0.29. In contrast, ODs of A2A2 defatted milk (n = 11) were all lower than the cut-off value and the mean OD was 0.12 ± 0.03 (Fig 2A). These findings demonstrated the specificity of the A1 mAb to the A1 but not A2 beta-casein.

Next, we applied the sandwich ELISA to raw milk, because the ELISA is expected to test milk collected in dairy farm. The OD values of the A1A1 (n = 4) and A1A2 (n = 13) raw milk were 1.19 ± 0.04 and 1.04 ± 0.11, respectively, which was all higher than the cut-off value (0.200). Consistently, the OD values of A2A2 (n = 18) raw milk was 0.17 ± 0.03, which was lower than the cut-off value (Fig 2B).

**Fig 3:**
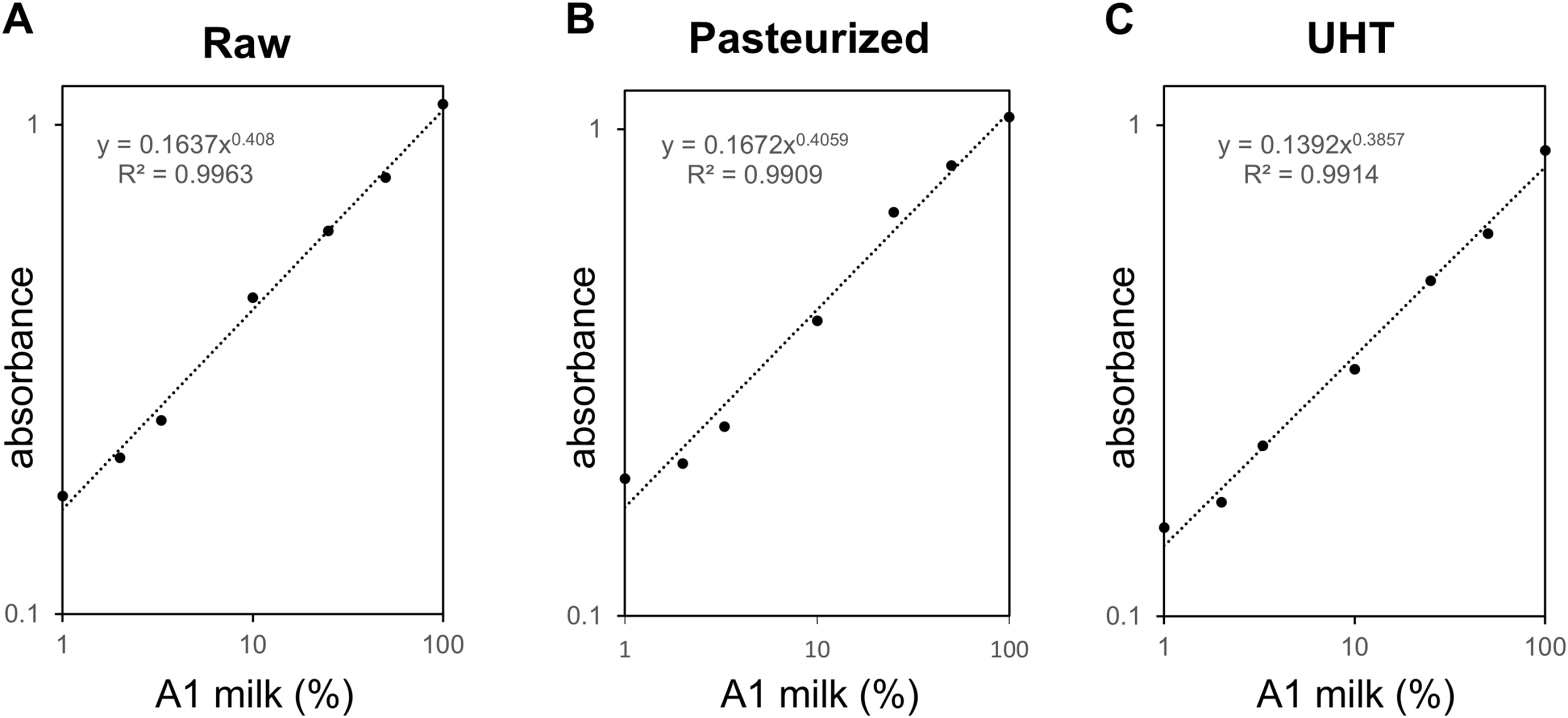
Standard curves in raw, pasteurized, and UHT milk. Standard curves in raw (A), pasteurized (B), and UHT milk (C). OD values of milk containing different spike levels of A1 (100%, 50%, 25%, 10%, 3%, 2%, 1%, and 0%) in A2 milk were the same as those shown in Table 1.

To determine whether an A1A1 milk spike could be detected in A2A2 milk with raw milk samples, we used mixtures of A1A1 at different ratios with A2A2 milk. The OD from negative control (pure A2 milk) was 0.15 ± 0.01. As the factor (*f*) was 1.82 at a 95% confidence level, the cut-off value was calculated as 0.16. As shown in Table 1, the OD values obtained from the mixtures with 50%, 25%, 10%, 3%, 2%, and 1% A1 spikes were higher than the cut-off value, indicating that these samples were positive for A1. The corresponding standard curve exhibited a high linearity ranging from 1% to 100% A1 spike and the correlation coefficient was > 0.99 (Fig 3A). These findings suggested that the sandwich ELISA could detect 1% or more A1A1 milk spike in A2A2 milk.

**Table 1:** Mean OD values obtained from raw, pasteurized, and UHT milk containing different spike levels of A1.

% A1 in A2	Raw		Pasteurized		UHT	
	OD^1^	CI^2^	OD	CI	OD	CI
100	1.10 ± 0.06	1.07–1.13	1.25 ± 0.13	1.19–1.32	0.84 ± 0.10	0.80–0.89
50	0.81 ± 0.08	0.77–0.85	0.93 ± 0.09	0.88–0.97	0.62 ± 0.04	0.60–0.64
30	0.69 ± 0.07	0.65–0.73	0.72 ± 0.05	0.70–0.75	0.49 ± 0.03	0.47–0.50
10	0.44 ± 0.05	0.42–0.47	0.46 ± 0.04	0.44–0.48	0.33 ± 0.03	0.31–0.35
3	0.29 ± 0.04	0.27–0.32	0.32 ± 0.08	0.28–0.37	0.26 ± 0.05	0.23–0.28
2	0.23 ± 0.03	0.21–0.24	0.23 ± 0.03	0.21–0.24	0.19 ± 0.03	0.17–0.20
1	0.18 ± 0.01	0.18–0.19	0.19 ± 0.02	0.18–0.20	0.16 ± 0.01	0.15–0.16
0	0.15 ± 0.01	0.14–0.15	0.14 ± 0.01	0.14–0.15	0.12 ± 0.004	0.11–0.12

^a^ Mean ± SD of OD values from 15 measurements.

^b^ Confidence interval with 95% confidence level.

We further attempted to detect A1 beta-casein in sterilized milk. The OD from negative control (pure A2 milk) was 0.15 ± 0.01 for pasteurized milk and 0.12 ± 0.004 for Ultra-high temperature (UHT) milk, respectively. As the factor (*f*) was 1.82 at a 95% confidence level, the cut-off value was calculated as 0.16 for pasteurized milk and 0.12 for UHT milk, respectively. As shown in Table 1 the 50%, 25%, 10%, 3%, 2%, and 1% A1 spike containing pasteurized and UHT mixtures showed larger OD values than the corresponding cut-off values, respectively. The corresponding standard curve exhibited a high linearity ranging from 1% to 100% A1 spike and the correlation coefficient was > 0.98 (Fig 3B and C). These findings also suggested that the sandwich ELISA could 1% or more A1A1 milk spike in the sterilized A2A2 milk.

## Discussion

In the present study, we developed a mAb specific to the bovine A1 beta-casein. We also developed a mAb that could react with both A1 and A2 beta-casein. The mAb against the beta-casein was used as the capture antibody and the A1-specific antibody was used as the detection antibody to develop a sandwich ELISA for detecting A1 beta-casein in milk.

The A1-specific mAb was raised with the peptide containing 67His. The sequence of it was CFPGPIH^67^NSLP and spans 61st-71st amino acids. Beta-casein variants (A1, A2, B, C, and A3) are defined by amino acids at 37, 67, 106, and 122 residues (Nomenclature of Proteins of Cow's Milk, 6th revision) [33]. Variants B and C share 67His with the A1 variant and are thought to react with the A1-specific antibody. A2 milk is defined by the amino acid at 67^th^ position. Thus, variants B and C are regarded as “A1” in the context of A2 milk and might release BCM-7.

The sandwich ELISA exhibited 100% sensitivity and 100% specificity when we tested a total of 67 genotype-known milk. Sensitivity indicates the probability of a positive result in truly positive samples and specificity is the probability of a negative result in negative samples [34]. Additionally, the ELISA detected A1 casein when A1A1 milk was present at a 1% spike level in A2A2 milk, suggesting that the detection level of the ELISA was 1% in volume. The ELISA was also applicable to sterilized milk, indicating that the epitopes recognized by the A1-specific mAb are preserved even after heat treatment. These results strongly suggest that the ELISA can be used to monitor A2 milk to be free A1 beta-casein before and after milk processing.

Although different proteomic methods with mass spectrometry have been developed to measure A1 and A2 variants in milk, most have not specifically quantified A1 in A2 milk. Notably, Wang et al. [22] developed an HPLC-MS/MS assay to quantify A1 and A2 beta-casein variants and reported detection limits of approximately 5% for both. An alternative approach is detecting the A1 allele using genetic methods such as quantitative PCR and rhAmp assays, which have achieved detection at a 2% spike level [35,36]. In comparison, the ELISA developed in this study can detect the A1 variant at a 1% spike level, suggesting that it offers greater sensitivity for quantifying A1 than existing genetic and proteomic assays.

To date, two anti-A1 antibodies have been available. One is a commercially available polyclonal anti-A1-IgY (Biosensis Pty Ltd, Thebarton, South Australia, Australia). This antibody has been used to develop an ELISA kit to detect A1 beta-casein in milk. The kit exhibited 100% sensitivity and 100% specificity [26] and could detect spikes of bovine A1A1 milk in goat and camel milk at 50%, 25%, and 5% spike level. Because the goat and camel milk are A2-type milk [37–39], Biosensis claims that the kit can detect A1 milk in A2 milk at a 5% or more spike level. Nonetheless, data with lower spike levels are not available. Moreover, the kit requires NaOH, a harmful reagent for human, to dilute milk samples. We found the kit failed to detect A1 milk spiked into A2 milk at levels below 50% when samples were diluted with PBS (S2 Table), which indicates that our sandwich ELISA assay exhibits superior sensitivity compared with the commercially available ELISA.

The other one is also a polyclonal anti–A1-IgY that was generated by de Jesus et al. [26]. The ELISA with the antibody could recognize the A1 beta-casein in cow's milk. However, the authors noticed one false-negative result with the ELISA system, indicating a sensitivity of 95.2%. Such discrepancy may be derived from the difference in the ELISA system. The ELISA of de Jesus et al. is the direct ELISA, in which the antibody is immobilized on the 96-well plate. Meanwhile, our ELISA is the sandwich ELISA, in which two different antibodies that recognize different epitopes are used. Thus, our ELISA system could be more sensitive and specific to the A1 beta-casein.

Our ELISA system was able to detect A1 beta-casein at a 1% spike level in both raw and sterilized milk, which was much lower than those observed in the other A1 beta-casein ELISA systems described above. This finding suggests that the sandwich ELISA can detect contamination from one A1A1 cow in a herd of one hundred A2A2 cows. In typical dairy farms, milk is collected from several dozens of cows and stored in a bulk tank. Thus, the sandwich ELISA system can offer enough ability to find contamination of A1 in A2 milk.

Although ELISA is recognized as economical and cost-effective method for the direct measurement of milk proteins such as beta-casein, potential sources of variability in the assay should be considered. Because ELISA detects A1 protein directly, results can vary depending on the total protein content in milk. Genetic and environmental factors, including breed differences [40], beta-lactoglobulin genotype and somatic cell counts [41], and seasonal variations [42] are known to affect milk protein composition and may therefore contribute to variability in assay results.

Despite its advantages over existing A1 beta-casein ELISA assays, our system has limitations. One major limitation is the lack of an A2-specific mAb, which prevents discrimination between A1A2 and A1A1 milk phenotypes. Additionally, we have not yet performed absolute quantification of A1 beta-casein in standard milk samples used to generate calibration curves. Further studies focusing on absolute quantification will help determine the precise amount of A1 variant that the ELISA can detect and improve its applicability for quality control.

Global A2 milk market size was estimated at 4.0 billion USD in 2024 and will reach 11.2 billion USD by 2030, with growing annually at 18.5% from 2025 to 2030 [22]. In such growing market, monitoring of A2 milk to be free from A1 is surely required. Indeed, mislabeling of A2 milk, the contamination of A1, was uncovered in market of Austria, which led costumer confusion [21].

The A1 beta-casein ELISA developed here can contribute to establishing a monitoring system for detecting A1 contamination in “A2 milk.” Such system would involve routine testing of raw bulk milk and sterilized milk at A2 milk production facilities, as well as ad hoc inspections of A2 milk products at retail outlets. When contamination with A1 is detected, the production plant can halt shipment and review its processing steps. These measures will help minimize unintentional mislabeling and enhance consumer safety. Furthermore, the A1-specific mAb could be adapted for a rapid and simple lateral flow immunoassay [25], making the inspection system more widely applicable and convenient.

In summary, we developed a mAb specific to the bovine A1 beta-casein and applied it to establish a novel sandwich ELISA assay for detecting A1 in milk. This assay was able to detect A1 at a 1% spike level in both raw and sterilized milk, which is substantially lower than the detection limits reported for other A1 beta-casein ELISA systems. With its higher sensitivity, non-hazardous nature, and stable performance, our ELISA assay can serve as a platform for monitoring A1 contamination in A2 milk, thereby contributing to quality control and consumer safety. Future improvements could include the generation of an A2-specific mAb, comparative evaluation with other protein detection methods, and development of simpler approaches such as lateral flow immunoassay. These advancements would make the system more rapid, widely applicable, and reliable.

## Supporting information

S1 Table BLASTP output of clusters producing significant alignments with the 67His peptide sequence. (PDF)

S2 Table OD values obtained from the commercially available A1 beta-casein ELISA kit. (PDF)

S3 Table Raw data sets to replicate all study findings reported in the article. (XLSX)

S1 Fig Raw images of gel and Western blots. (PDF)
