# Supplementary material for: Development of a new sandwich ELISA for the detection of bovine A1 beta-casein
Source: PLoS One. 2026 Apr 9;21(4):e0345548. doi: 10.1371/journal.pone.0345548 (PMC13065063; doi:10.1371/journal.pone.0345548)
Supplement: S1 Table — (PDF) [file pone.0345548.s001.pdf]

S1 Table. BLASTP output of clusters producing significant alignments with the 67His peptide sequence.

Representative sequence	Max Score	Total Score	Query Cover	E value	Per. ident	Acc. Len	Accession
beta casein B, partial [Bos taurus]	35.8	35.8	91%	9.00E-05	100	146	CAC37028.1
beta-casein precursor, partial [Bos taurus]	30.3	42.8	91%	0.008	90	119	AAW84270.1
kappa-casein, partial [Bos taurus][a]	30.3	42.8	91%	0.008	90	105	AAW84271.1
beta-casein, partial [Bos taurus]	30.3	30.3	91%	0.008	90	38	AAB59254.1
beta-casein, partial [Bos taurus]	26.5	26.5	91%	0.16	80	24	QYD13589.1
beta-casein, partial [Bubalus bubalis]	26.5	26.5	91%	0.19	80	82	QPB17913.1
beta-casein precursor [Ovis aries]	25.2	25.2	91%	0.53	80	222	NP_001009373.1
beta-casein, partial [Capra hircus]	25.2	25.2	91%	0.53	80	160	QHN12642.1

[a]The third row of the results appears to list κ-casein; however, the associated sequence (ACC# AAW84271) is likely misannotated, as BLASTP confirmed that it shares significant similarity with bovine β-casein.
