# Supplementary material for: Development of a new sandwich ELISA for the detection of bovine A1 beta-casein
Source: PLoS One. 2026 Apr 9;21(4):e0345548. doi: 10.1371/journal.pone.0345548 (PMC13065063; doi:10.1371/journal.pone.0345548)
Supplement: S2 Table — (PDF) [file pone.0345548.s002.pdf]

S2 Table. OD values obtained from the commercially available A1 beta-casein ELISA kit.

% A1in A2	OD
100	0.128
50	0.097
25	0.084
10	0.075
3	0.076
2	0.08
1	0.075
0	0.068
