# Supplementary material for: Development of a new sandwich ELISA for the detection of bovine A1 beta-casein
Source: PLoS One. 2026 Apr 9;21(4):e0345548. doi: 10.1371/journal.pone.0345548 (PMC13065063; doi:10.1371/journal.pone.0345548)
Supplement: S1 Fig — (PDF) [file pone.0345548.s004.pdf]

15% SDS-PAGE and stained with Coomassie Brilliant Blue

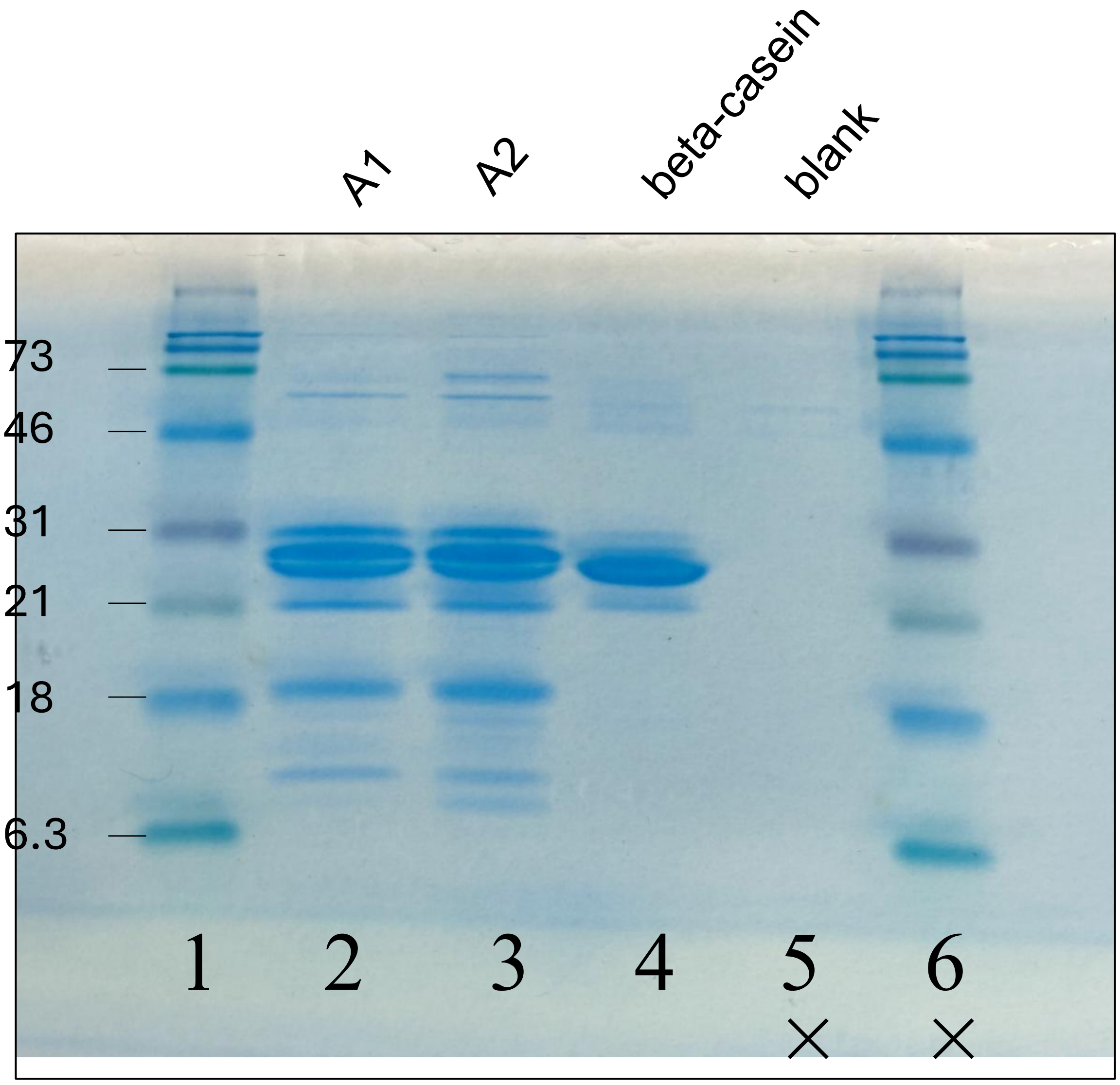

1. Molecular marker
2. Milk protein extracts (4.2 µg) from A1A1 samples
3. Milk protein extracts (4.2 µg) from A2A3 samples
4. The purified beta-casein (4.2µg)
5. Blank
6. Molecular marker

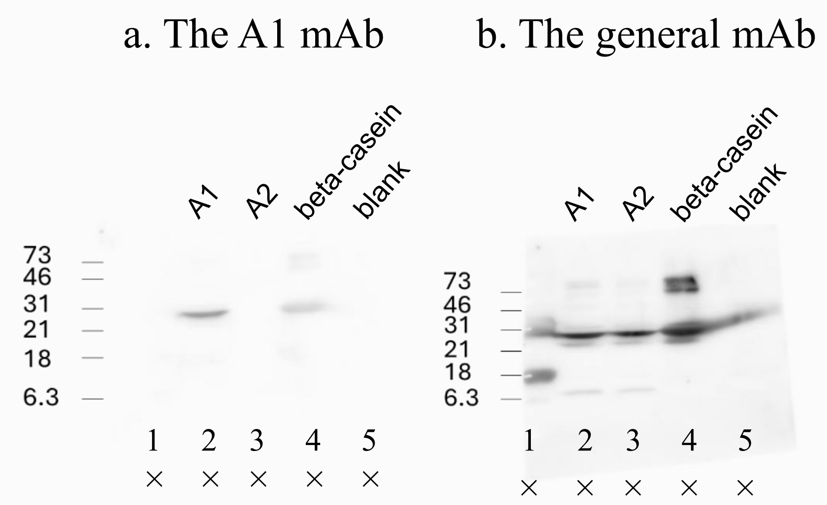

1. Molecular marker
2. Milk protein extracts (4.2 μg) from A1A1 samples
3. Milk protein extracts (4.2 μg) from A2A3 samples
4. The purified beta-casein (4.2μg)
5. blank

Exposure time 10 sec

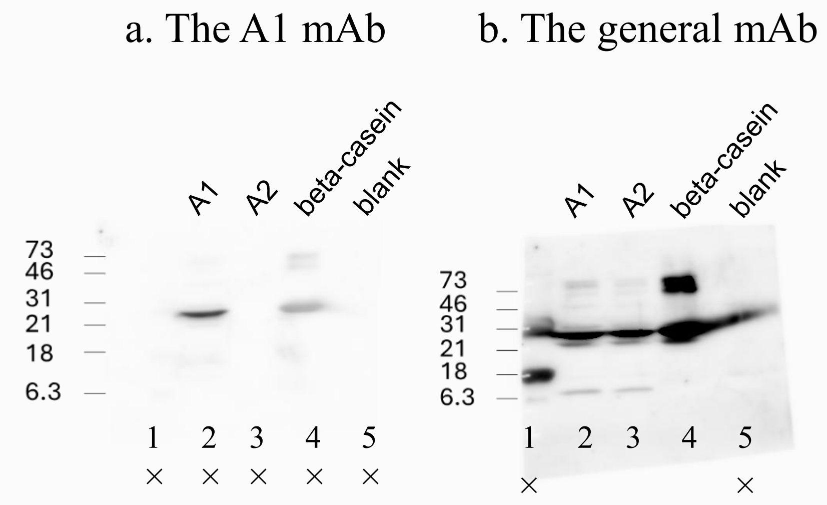

1. Molecular marker
2. Milk protein extracts (4.2 μg) from A1A1 samples
3. Milk protein extracts (4.2 μg) from A2A3 samples
4. The purified beta-casein (4.2μg)
5. blank

Exposure time 20 sec

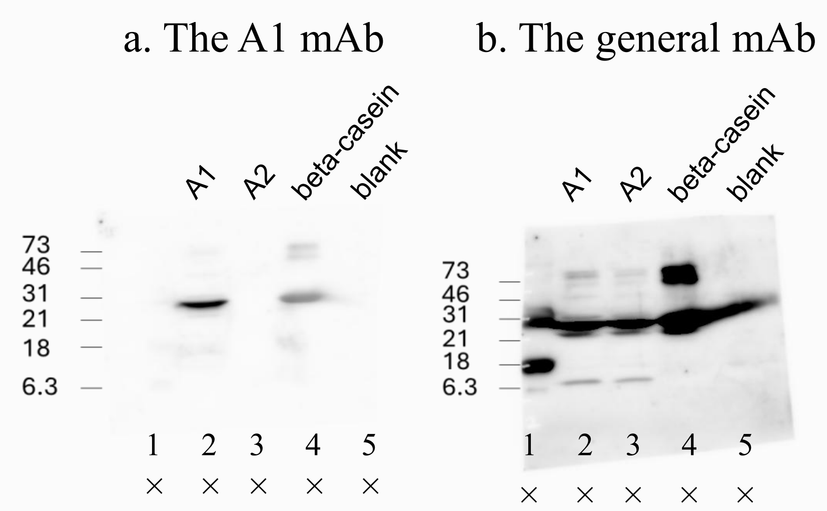

1. Molecular marker
2. Milk protein extracts (4.2 μg) from A1A1 samples
3. Milk protein extracts (4.2 μg) from A2A3 samples
4. The purified beta-casein (4.2μg)
5. blank

Exposure time 30 sec

a. The A1 mAb　　b. The general mAb

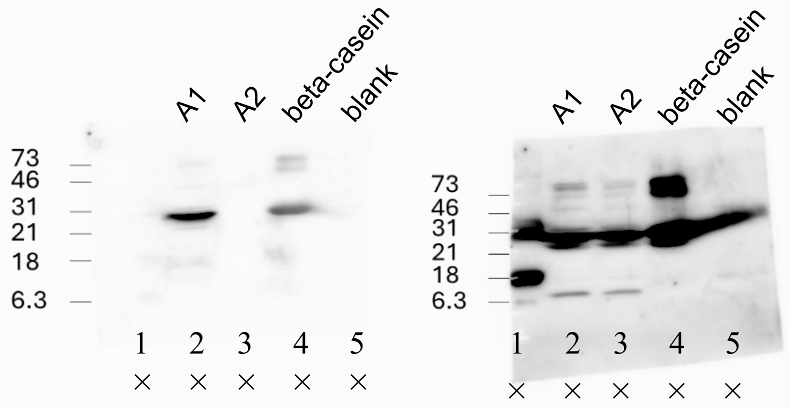

1. Molecular marker
2. Milk protein extracts (4.2 µg) from A1A1 samples
3. Milk protein extracts (4.2 µg) from A2A3 samples
4. The purified beta-casein (4.2µg)
5. blank

Exposure time 40 sec

a. The A1 mAb　　b. The general mAb

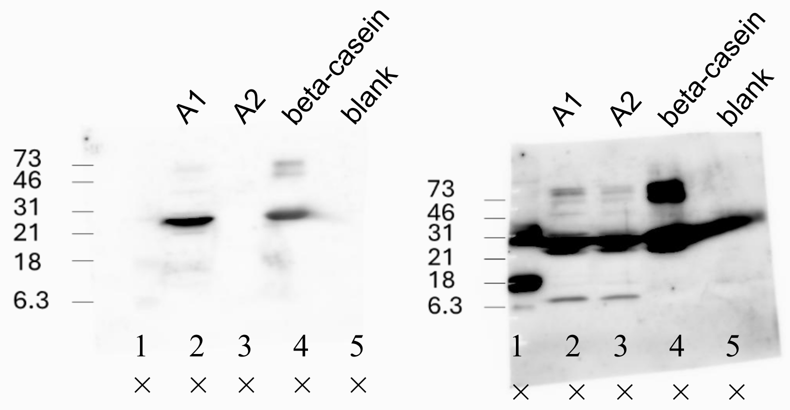

1. Molecular marker
2. Milk protein extracts (4.2 µg) from A1A1 samples
3. Milk protein extracts (4.2 µg) from A2A3 samples
4. The purified beta-casein (4.2µg)
5. blank

Exposure time 50 sec

a. The A1 mAb　　b. The general mAb

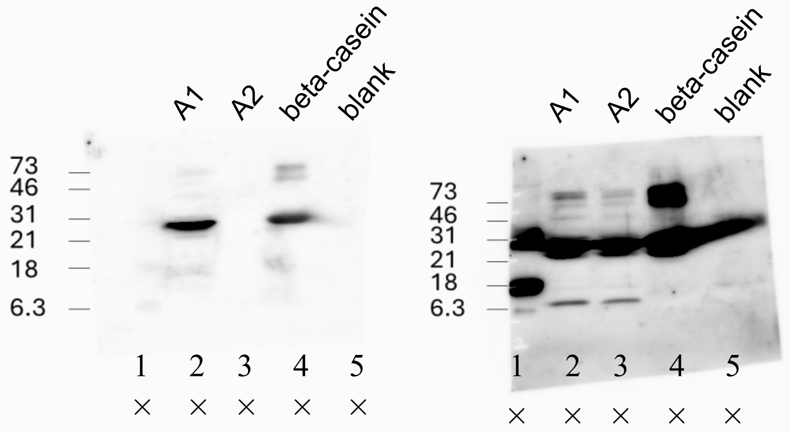

1. Molecular marker
2. Milk protein extracts (4.2 µg) from A1A1 samples
3. Milk protein extracts (4.2 µg) from A2A3 samples
4. The purified beta-casein (4.2µg)
5. blank

Exposure time 60 sec

a. The A1 mAb　　b. The general mAb

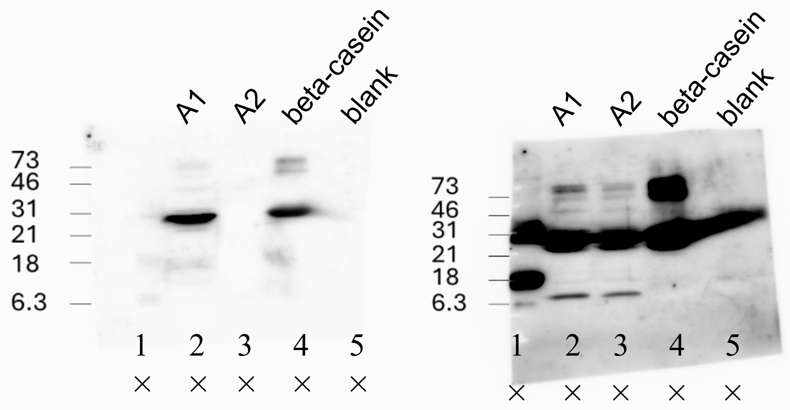

1. Molecular marker
2. Milk protein extracts (4.2 μg) from A1A1 samples
3. Milk protein extracts (4.2 μg) from A2A3 samples
4. The purified beta-casein (4.2μg)
5. blank

Exposure time 70 sec

a. The A1 mAb　　b. The general mAb

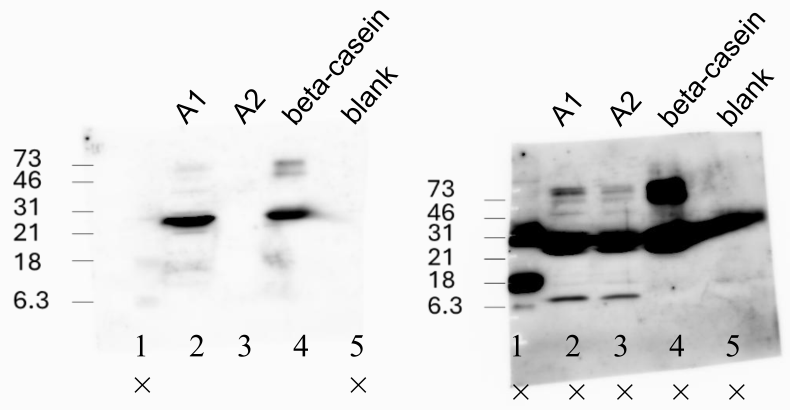

1. Molecular marker
2. Milk protein extracts (4.2 μg) from A1A1 samples
3. Milk protein extracts (4.2 μg) from A2A3 samples
4. The purified beta-casein (4.2μg)
5. blank

Exposure time 80 sec
